# Lattice Symmetry‐Guided Charge Transport in 2D Supramolecular Polymers Promotes Triplet Formation

**DOI:** 10.1002/advs.202402932

**Published:** 2024-06-12

**Authors:** Ruggero Emmanuele, Hiroaki Sai, Jia‐Shiang Chen, Darien J. Morrow, Luka Đorđević, David J. Gosztola, Saw Wai Hla, Samuel I. Stupp, Xuedan Ma

**Affiliations:** ^1^ Center for Nanoscale Materials Argonne National Laboratory Lemont IL 60439 USA; ^2^ Simpson Querrey Institute for BioNanotechnology Northwestern University Chicago IL 60611 USA; ^3^ Center for Molecular Quantum Transduction Northwestern University Evanston IL 60208 USA; ^4^ Department of Chemistry Northwestern University Evanston IL 60208 USA; ^5^ Nanoscale and Quantum Phenomena Institute and Department of Physics and Astronomy Ohio University Athens OH 45701 USA; ^6^ Department of Materials Science and Engineering Northwestern University Evanston IL 60208 USA; ^7^ Department of Medicine Northwestern University Chicago IL 60611 USA; ^8^ Consortium for Advanced Science and Engineering University of Chicago Chicago IL 60637 USA

**Keywords:** fourier imaging, lattice symmetry, Monte Carlo simulations, spin‐uncorrelated charge carriers, supramolecular polymers, triplets

## Abstract

Singlet‐to‐triplet intersystem crossing (ISC) in organic molecules is intimately connected with their geometries: by modifying the molecular shape, symmetry selection rules pertaining to spin‐orbit coupling can be partially relieved, leading to extra matrix elements for increased ISC. As an analog to this molecular design concept, the study finds that the lattice symmetry of supramolecular polymers also defines their triplet formation efficiencies. A supramolecular polymer self‐assembled from weakly interacting molecules is considered. Its 2D oblique unit cell effectively renders it as a coplanar array of 1D molecular columns weakly bound to each other. Using momentum‐resolved photoluminescence imaging in combination with Monte Carlo simulations, the study found that photogenerated charge carriers in the supramolecular polymer predominantly recombine as spin‐uncorrelated carrier pairs through inter‐column charge transfer states. This lattice‐defined recombination pathway leads to a substantial triplet formation efficiency (≈60%) in the supramolecular polymer. These findings suggest that lattice symmetry of micro‐/macroscopic structures relying on intermolecular interactions can be strategized for controlled triplet formation.

## Graphical abstract

To overcome moderate triplet formation efficiencies in chromophores, molecular geometry is utilized to relieve symmetry selection rules and increase intersystem crossing. Here, symmetry is discovered to play a similar role in supramolecular structures. By constructing a supramolecular polymer with an asymmetric unit cell, the study found that triplet formation efficiency is increased to 60% from 1% in the constituting molecules.

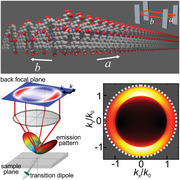

## 1 Introduction

Since many organic chromophores have singlet ground states and weak spin‐orbit coupling, their photogenerated triplets are typically long‐lived. This very attribute of triplets has prompted various applications based on them, including photocatalytic reactions utilizing triplet sensitizers,^[^ ^1^, ^2^ ^]^ photon up‐conversion via triplet‐triplet annihilation,^[^ ^3^, ^4^ ^]^ and blue light‐emitting diodes with long lifetimes.^[^ ^5^ ^]^ However, due to the modest intersystem crossing (ISC) in these organic chromophores, triplet formation during optical excitation is extremely inefficient.^[^ ^6^, ^7^ ^]^ To mitigate the low formation efficiencies of triplets in organic molecules without invoking the heavy‐atom effect that sacrifices the long lifetimes of triplets, synthetic chemists have successfully developed molecular design strategies that exploit geometry to break spin‐orbit coupling symmetry selection rules to increase ISC rates. For instance, compact electron donor‐acceptor dyads, in which the donor and acceptor are directly linked to one another in an *orthogonal* geometry, have highly efficient ISC due to the orbital angular momentum changes during charge recombination.^[^ ^8^, ^9^ ^]^ Another elegant strategy that leverages geometry for enhanced ISC is the use of a *twisted* π‐conjugated framework, in which the broken mirror symmetry induces additional spin‐orbit coupling matrix elements.^[^ ^10^, ^11^, ^12^ ^]^

Moving beyond individual molecules, highly ordered supramolecular polymers can be self‐assembled from corresponding monomers through noncovalent interactions. A particularly intriguing feature of these supramolecular polymers is that they are held together by weak intermolecular bonds, the nature and strength of which can be edited by the structures of the monomers and the local environment. The versatile nature of supramolecular polymers provides the possibility to design and engineer them in various ways for multifunctional purposes. As such, supramolecular polymers have been utilized for photocatalysis,^[^ ^13^, ^14^ ^]^ solar energy conversion,^[^ ^15^ ^]^ and flexible electronics,^[^ ^16^, ^17^, ^18^ ^]^ to name a few.

In many cases, the idea of structures that have been successful in describing molecules has also been proven to be successful in describing supramolecular structures. This perspective has greatly benefited the progress of the supramolecular field.^[^ ^19^ ^]^ In the same way that a molecule can be defined as an assembly of atoms, a supramolecular structure can be viewed as orderly packed molecules held together by weak bonds. With this analogy in mind, we explore and identify a design concept that exploits the geometry of supramolecular structures defined by their molecular arrangements for efficient triplet generation. Specifically, we consider self‐assembled 2D supramolecular polymers consisting of loosely bonded coplanar 1D molecular columns. This geometry results in photogenerated charge carriers preferentially transporting along the 1D molecular columns. Using the Fourier imaging technique, we discover a peculiar in‐plane emission dipole lying perpendicularly to the charge transport direction. Combining static and time‐resolved optical studies with Monte Carlo simulations, we assign this emission dipole to singlet charge transfer states (^1^CT_inter_) formed by spin‐uncorrelated electrons and holes residing in neighboring molecular columns within the same supramolecular polymer. The encounters of these spin‐uncorrelated charge carriers also lead to a substantial population of spin triplets (^3^CT_inter_ ≈ 60%), the subsequent downhill relaxation of which leads to efficient triplet formation. These results suggest that the design concept of using molecular geometry to promote ISC can be extended analogously to their supramolecular self‐assemblies. The arrangement of monomers and the resultant geometry can determine exciton spin states and charge recombination pathways in supramolecular structures.

## 2 Results and Discussion

### 2.1 Structural and Optical Properties of the Supramolecular Nanoribbons

**Figure 1:**
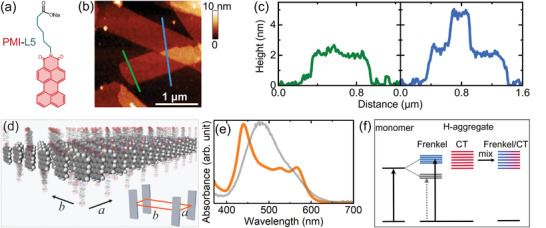
a) Molecular structure of the PMI‐L5 molecules. b) Atomic force microscopy image of the supramolecular nanoribbons. c) Height profiles extracted from (b) indicate that the nanoribbons are around 2 nm thick. d) Illustration of the supramolecular nanoribbons and their unit cells. e) Absorption spectra of the PMI‐L5 molecules (gray) and nanoribbons (orange) in water. f) Schematic of the charge‐transfer/Frenkel mixing in the supramolecular nanoribbons.

Supramolecular polymers used in this study are constructed from chromophore amphiphiles containing a perylene monoimide (PMI) light‐absorbing segment covalently attached to a carboxylate headgroup (referred to as PMI‐L5, **Figure** **1**a). These amphiphiles, upon dissolution and annealing under charge‐screening aqueous conditions (see Experimental Section for details), self‐assemble spontaneously into ribbon‐like crystalline supramolecular polymers that are typically 0.5–3 µm wide, ≈10 µm long, and one molecular layer (≈2 nm) thick (Figure 1b,c). The resultant crystalline structures adapt a 2D oblique unit cell with intermolecular distances of *a* = 3.6 Å and *b* = 8.3 Å (Figure 1d).^[^ ^20^, ^21^ ^]^ This structure is mostly defined by the distinct natures of molecular interactions along the two axes: along the *a*‐axis, which runs parallel to the length of the supramolecular nanoribbons,^[^ ^21^ ^]^ the molecules stack closely in a face‐to‐face manner, forming 1D molecular columns through a combination of π–π and dipolar interactions, while along the *b*‐axis (width of the nanoribbons), the 1D columns are linked to neighboring columns through weak dipolar interactions.^[^ ^22^ ^]^

Figure 1e shows the absorption spectra of PMI‐L5 molecules and supramolecular nanoribbons in water. Compared to the absorption spectrum of the PMI‐L5 molecules, the supramolecular nanoribbons exhibit two major peaks, one at 440 nm and the other at 570 nm, bridged by a broad continuous absorption band. This spectral feature of the nanoribbons and the corresponding electronic structures are intimately related to their molecular arrangements.^[^ ^20^, ^23^ ^]^ Specifically, the co‐facial π–π stacking of the molecules along the *a*‐axis leads to substantial overlaps of the adjacent molecules’ frontier molecular orbitals and consequently, strong intermolecular interactions within the 1D molecular columns. The high dielectric environment of the supramolecular polymers further promotes extended charge‐transfer states (referred to as ^1^CT_intra_) with large electron‐hole separations along the *a*‐axis. Coupling of these extended charge‐transfer states with molecular Frenkel excitations leads to a band of optically active states (Figure 1f), which are manifested as the broad absorption spectra of the supramolecular nanoribbons.

### 2.2 Optical Transition Dipoles of the Supramolecular Nanoribbons

**Figure 2:**
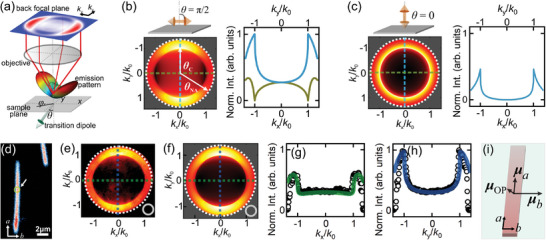
a) A sketch illustrating the basic concept of back focal plane (BFP) imaging. b,c) Left: Numerically simulated BFP images of a linear dipole lying horizontally on (b) and perpendicularly to (c) the sample plane. Right: Line profiles along the *k*_x_ (green) and *k*_y_ (blue) directions. d) A real‐space photoluminescence image of supramolecular nanoribbons deposited on substrates. The *a*‐ and *b*‐axes of the nanoribbon used for BFP imaging are indicated. The dashed circle indicates the position on the nanoribbon where photoluminescence was collected for BFP images in (e). e,f) BFP images measured from the area indicated in (d) e) and numerically simulated using the transition dipole strength ratio of |*µ_a_* |^2^: |*µ_b_* |^2^: |*µ*_OP_|^2^ = 6.0:11.6:1.0 f). The gray circles represent that no polarization selection was performed to the emission. g,h) Line profiles extracted from the experimentally measured BFP image in (e) (circles) and the numerically simulated image in (f) (solid lines). i) An illustration of the three transition dipole components in the nanoribbons.

Bearing these electronic and structural properties of the supramolecular nanoribbons in mind, we deploy a Fourier imaging technique^[^ ^24^, ^25^, ^26^ ^]^ to investigate the charge recombination pathways in the system. As illustrated in **Figure** **2**a, the Fourier imaging approach detects momentum‐resolved emission patterns from individual nanoribbons at the back focal plane (BFP) of a home‐built confocal laser microscope (see Experimental Section for details). It allows us to determine the 3D orientations of emission transition dipoles in the supramolecular nanoribbons. The emission transition dipole, ***µ***, of a supramolecular nanoribbon can be considered as a superposition of three orthogonal transition dipole components (Figure 2i): one perpendicular to the sample plane (***µ***_OP_), the other two in‐plane and along the *a*‐ and *b*‐axes of the nanoribbon (***µ***_a_ and ***µ***_b_). Using an analytical model,^[^ ^24^ ^]^ the BFP images can be well described by: $I^{s,p}\left(k_{x},k_{y}\right)=\eta C_{0}\sum_{l=a,\;b,\;\mathrm{OP}}{|\mu_{l}|}^{2}{\tilde{\rho }}_{l}^{s,p}\left(k_{x},k_{y}\right)$, where *s* and *p* denote polarizations of the electromagnetic wave, η*C*_0_ is a proportional constant related to measurement conditions and light frequencies, ${\tilde{\rho }}_{a,\;b,\;\mathrm{OP}}^{s,p}$ is the normalized local density of optical states for the three orthogonal emission dipole components, and | ***μ***_a, b,  OP_|^2^ is the dipole strength of the three emission dipole moment components (see Section S1, Supporting Information for details). As such, the *k*‐vector resolved emission patterns obtained at the BFP are highly sensitive to the relative strength ratios of ***µ***_OP_, ***µ*** *_a_*, and ***µ*** *_b_*, which together define the 3D orientation of the emission transition dipole, ***µ***.

Figure 2b shows a simulated BFP image of an in‐plane transition dipole (*θ = *π/2) using the abovementioned model. Two distinct regions can be observed in the BFP image, with their boundary corresponding to the critical angle (*θ*_C_) of total internal reflection at the glass‐air interface.^[^ ^26^ ^]^ The outer circle is defined by the maximum collection angle of the objective (*θ*_NA_) used in this study. The simulated BFP image of an out‐of‐plane transition dipole lying perpendicularly to the sample plane (*θ = *0) shows similar inner and outer regions, but an apparently different emission pattern (Figure 2c). More details are revealed in the line cuts of the BFP images (Figure 2b,c, right columns), with the most notable features being: i) the different intensity ratios between the extrema of the line cuts along *k*_x_ and *k*_y_, and ii) the distinct intensities at the *k*_x_(*k*_y_) → 0 regions. We primarily rely on these two factors for deriving the relative strength ratios of the three orthogonal dipole components.

**Figure 3:**
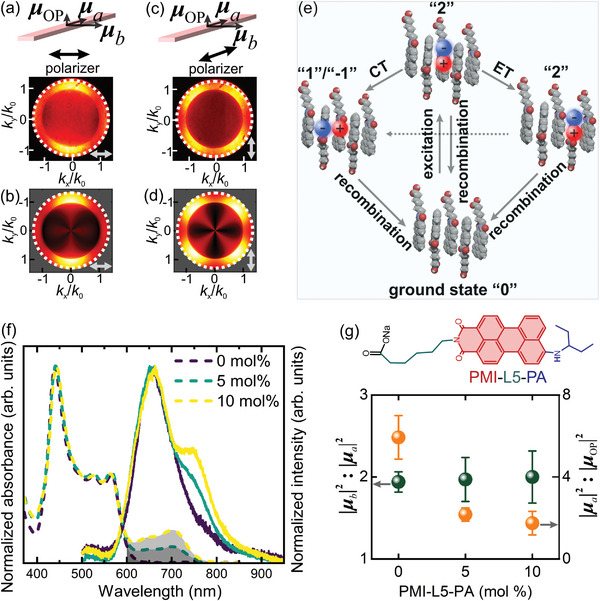
a,b) Experimentally measured a) and numerically simulated b) BFP images of a supramolecular nanoribbon with a linear polarizer aligned parallel to the *b*‐axis of the nanoribbon and placed in the detection beam path. c,d) Experimentally measured c) and numerically simulated d) BFP images of the same supramolecular nanoribbon with a linear polarizer aligned parallel to the *a*‐axis of the nanoribbon and placed in the detection beam path. e) A sketch illustrating the processes that an optically excited Frenkel exciton can undergo. ET: energy transfer; CT: charge transfer. f) Normalized absorption (dashed) and emission (solid) spectra of supramolecular nanoribbons integrated with 0–10 mol% of PMI‐L5‐PA molecules. As the concentration of PMI‐L5‐PA increases, an additional broad peak at ≈600–700 nm appears in the absorption spectra (gray area), which originates from the PMI‐L5‐PA molecules. g) Molecular structure of the PMI‐L5‐PA molecule (top) and its influence on the ratios among the three transition dipole components (bottom).

Figure 2d shows a real‐space photoluminescence (PL) image of a nanoribbon on a substrate. Its BFP image (Figure 2e) and line cuts along the *a*‐ and *b*‐axes (Figure 2g,h, circles) reveal an emission dipole strength ratio of | ***µ*** *_a_* |^2^: | ***µ*** *_b_* |^2^: | ***µ***_OP_|^2^ = 6.0: 11.6: 1.0 when analyzed using the abovementioned model (see Figure 2g,h for fitting results, and Figure 2f for a simulated BFP image using the derived dipole strength ratio). Similar dipole strength ratios are obtained when repeating such measurements and analyses on more than 20 nanoribbons, with the in‐plane dipole strength along the nanoribbon width (| ***µ*** *_b_* |^2^) being twice of that along the length (| ***µ*** *_a_* |^2^), and the out‐of‐plane dipole strength (| ***µ***_OP_|^2^) considerably smaller than the in‐plane ones (Figure 2i). These results are further confirmed by performing polarization‐dependent BFP imaging, where emission along the nanoribbon width and length is selectively probed (**Figure** **3**a–d). A good agreement between the experimental and simulation results is obtained.

The strength ratio among the three emission dipole components reveals rich information about the carrier dynamics in the supramolecular nanoribbons. Upon photoexcitation (Figure 3e), optically allowed Frenkel excitons with their transition dipole moments oriented parallel to the PMI molecules^[^ ^27^ ^]^ (hence perpendicular to the nanoribbon surface defined by the *a*‐ and *b*‐axes, namely out‐of‐plane) are generated. There are several pathways for the Frenkel excitons to return to the ground state: they can i) decay, giving rise to the out‐of‐plane dipole component, ***µ***_OP_, ii) propagate to adjacent molecules through resonant energy transfer, or iii) dissociate primarily within the molecular columns and populate the charge‐transfer states (^1^CT_intra_) through the efficient charge‐transfer/Frenkel exciton mixing. The latter process leads to spatially separated electrons and holes along the 1D molecular columns, the recombination of which results in an emission transition dipole along the *a*‐axis of the nanoribbons, ***µ*** *_a_*. The | ***µ*** *_a_* |^2^: | ***µ***_OP_|^2^ = 6.0: 1.0 ratio obtained from the BFP images suggests more dominant emission from the ^1^CT_intra_ states than from the Frenkel excitons, consistent with previous studies predicting that in‐plane charge‐transfer states in a 1D molecular column would carry the majority of the oscillator strength.^[^ ^23^ ^]^

The assignment of ***µ***_OP_ and ***µ*** *_a_* is further confirmed by adjusting the compositions of the supramolecular nanoribbons. Adapting a recently developed method,^[^ ^28^ ^]^ we modify the PMI‐L5 molecule by decorating it with an additional 3‐pentylamino group (called PMI‐L5‐PA, Figure 3g top) and alloy this new molecule to the supramolecular nanoribbons (see Experimental Section for details). Due to the low density of the PMI‐L5‐PA molecules in the nanoribbons and their similar molecular structures to PMI‐L5, their integration in the nanoribbons does not have a noticeable effect on the crystalline structures of the supramolecular nanoribbons. The PMI‐L5‐PA molecules are chosen because their absorption (≈600–700 nm) overlaps with the emission of the nanoribbons (Figure 3f).^[^ ^28^ ^]^ As such, the PMI‐L5‐PA molecules serve as charge carrier “sinks,” leading to a reduction in the charge‐transfer carriers in the nanoribbons and their associated emission. Precisely due to this, we observe an apparent reduction in the | ***µ*** *_a_* |^2^: | ***µ***_OP_|^2^ ratio as the concentration of PMI‐L5‐PA molecules in the nanoribbons increases (Figure 3g, orange), which further confirms the charge‐transfer origin of ***µ*** *_a_*.

### 2.3 Monte Carlo Simulations and In‐Plane Charge Recombination Pathways

While charge transfer states resulting from dissociation of the Frenkel excitons along the 1D molecular columns (^1^CT_intra_) can well account for the dipole component along *a*‐axis, ***µ*** *_a_*, the strongest emission dipole component, ***µ*** *_b_*, observed in the BFP images cannot be related to any direct optical excitations. Specifically, due to the large intermolecular distance along *b*‐axis and small overlap between the frontier molecular orbitals of molecules in neighboring molecular columns, a direct creation of charge carriers along *b*‐axis through charge‐transfer/Frenkel mixing is negligible.^[^ ^23^ ^]^ These structural features of the supramolecular nanoribbons suggest an indirect generation mechanism of the emission transition dipole, ***µ*** *_b_*.

To identify the origin of this prominent emission dipole component, ***µ*** *_b_*, we perform kinetic Monte Carlo simulations to examine the charge carrier dynamics. The supramolecular nanoribbons are represented by a 2D rectangular lattice. Once generated (Figure 3e), the initial Frenkel excitation “2” can i) decay back to the ground state “0”, ii) undergo energy transfer to an adjacent molecule, or iii) participate in charge transfer provided its neighboring molecule is in the ground state, resulting in an electron (hole) in the original molecule and a hole (electron) in the neighbor. We label the charge‐transfer generated electrons and holes as “−1” and “+1”, respectively. These charge carriers can continue to hop to an empty neighbor or recombine upon the encounter of a counter charge. The probability for a Frenkel exciton or a charge carrier hopping from site *i* to one of its four neighbors, *j*, in a given time interval δ*t*, is given by:^[^ ^29^ ^]^

(1) $$P_{i-j}=\left[1-\exp\left(-\delta t\right)\right]\frac{\Gamma_{i\to j}}{\sum_{k=1,\text{…},4}\Gamma_{i\to k}}$$

Here, we adapt the Miller‐Abrahams model^[^ ^30^, ^31^ ^]^ to describe the intermolecular hopping rate Γ_i → j_ = Γ_0_exp(− β*d*), with Γ_0_ being the attempt‐to‐hop rate Γ_ET_ or Γ_CT_, *d* the intermolecular spacing (3.6 Å for intra‐column and 8.3 Å for inter‐column), and $\beta =\sqrt{2m\Delta E/\hbar^{2}}$ a normalizing tunnelling decay parameter^[^ ^32^ ^]^ that accounts for the hopping driving energy, Δ*E*, of the Frenkel or charge‐transfer excitons, and *m* the effective masses of the excitons and charge carriers (see Experimental Section for details).

**Figure 4:**
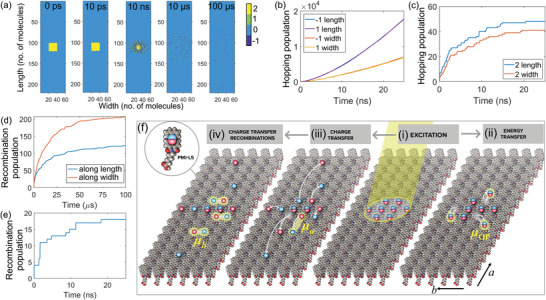
a) Evolutions of Frenkel excitons (“2”, yellow) and charge carriers (“1” and “−1″, green and blue) in a 220 × 60 2D lattice at various time delays after the initial optical excitation at time *t* = 0 s. b,c) Total number of hops made by the charge carriers b) and Frenkel excitons c) along the length (*a*‐axis) and width (*b*‐axis) of the 2D lattice till certain time after the initial excitation. d,e) Populations of charge carriers d) and Frenkel excitons e) undergoing recombination along the length (*a*‐axis) and width (*b*‐axis) of the 2D lattice. f) Schematics of the photophysical processes in the supramolecular nanoribbons upon optical excitation: within their lifetimes, the photogenerated Frenkel excitons i) can undergo energy transfer ii) or dissociate into charge carriers primarily along the 1D molecular columns, the subsequent recombination of which gives rise to the intra‐column transition dipole, ***µ*** *_b_* iii). In parallel, spin‐uncorrelated charge carriers in adjacent molecular columns can recombine, resulting in the inter‐column transition dipole, ***µ*** *_b_*, and triplet excitons iv).

Snapshots of the system at various times after the initial excitation (**Figure** **4**a) reveal a rapid reduction in the Frenkel exciton population due to their recombination and dissociation, and a correlated buildup of charge carrier populations (Figure S2, Supporting Information). Tracking the populations of charge carriers and Frenkel excitons that hop from one molecule to an available neighbor, we observe that they, especially the charge carriers, preferentially move along the length (or *a*‐axis) of the nanoribbons (Figure 4b,c), which is expected due to the smaller intermolecular distance in this direction. Interestingly, counterintuitive to the higher mobilities of the charge carriers along the *length* direction, their recombination, which is a direct reflection of the two in‐plane emission transition dipoles (***µ*** *_a_* along length and ***µ*** *_b_* along width), occurs more frequently along the *width* direction (Figure 4d). The recombination events along the length and width converge to a constant ratio of ≈1:2 by the time that most of the populations have decayed back to the ground state. In addition, the Frenkel exciton recombination, which is responsible for the out‐of‐plane dipole (***µ***_OP_), has an overall occurrence probability much smaller compared to the in‐plane events (Figure 4e). Together, we derive a simulated strength ratio of | ***µ*** *_a_* |^2^: | ***µ*** *_b_* |^2^: | ***µ***_OP_|^2^ = 6.8: 11.0: 1.0. These values are in good agreement with the | ***µ*** *_a_* |^2^: | ***µ*** *_b_* |^2^: | ***µ***_OP_|^2^ = 6.0: 11.6: 1.0 we obtain from the BFP images.

The Monte Carlo simulations suggest that the emission transition dipole along the nanoribbon width, ***µ*** *_b_*, originates from the recombination of charge carriers spatially separated in neighboring molecular columns. Due to the molecular packing arrangement in the supramolecular nanoribbons, efficient Frenkel exciton dissociation and long‐range charge separation occur predominantly along the 1D molecular columns (up to tens of molecules from previous theoretical studies^[^ ^23^ ^]^). As a result, intra‐column recombination of the charge carriers is expected to be slow due to the high mobilities of the charge carriers and the resultant large electron‐hole separation distances (Figure 4f,iii). These exact characteristics of the charge carriers, on the other hand, give rise to higher encounter and recombination probabilities of carriers that are in adjacent 1D molecular columns (Figure 4f,iv). Bimolecular recombination of these *spin‐uncorrelated* charge carriers spatially separated in neighboring molecular columns can result in singlet (^1^CT_inter_) and triplet (^3^CT_inter_) charge‐carrier states in a 1:3 ratio according to spin statistics.^[^ ^33^ ^]^ The singlet ^1^CT_inter_ state can decay back to the ground state radiatively, giving rise to the inter‐column emission transition dipole, ***µ*** *_b_*, or nonradiatively. From | ***µ*** *_a_* |^2^: | ***µ*** *_b_* |^2^ = 6.0: 11.6, we can derive that the inter‐column ^1^CT_inter_ state recombines more efficiently than the intra‐column ^1^CT_intra_ state, likely due to the larger charge separation distance of the ^1^CT_intra_ state.^[^ ^13^, ^23^, ^34^ ^]^ The inter‐column charge carrier recombination origin of ***µ*** *_b_* is also consistent with the constant | ***µ*** *_a_* |^2^: | ***µ*** *_b_* |^2^ ratio observed in Figure 3g upon an increase in the PMI‐L5‐PA concentration, because the PMI‐L5‐PA molecules reduce the overall charge carrier populations, rather than their inter‐/intra‐column recombination probabilities.

**Figure 5:**
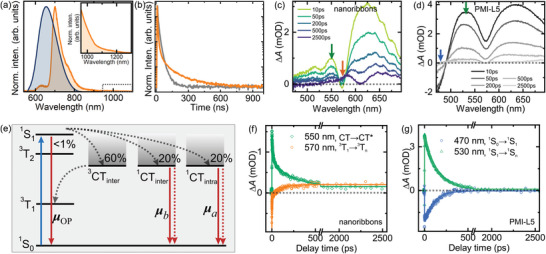
a) Photoluminescence (PL) spectra of the nanoribbons measured at room temperature (blue) and 5 K (orange). b) Time‐resolved PL measurements of PMI‐L5 molecules (gray) and nanoribbons (orange) measured at 5 K. c,d) Transient spectra of nanoribbons c) and PMI‐L5 molecules d) measured at room temperature. e) Schematics of the energy levels and transition dipoles involved in the study. The solid and dashed red lines represent radiative and nonradiative recombination, respectively. f,g) Transient kinetics of nanoribbons f) and PMI‐L5 molecules g) measured at room temperature. Solid lines are exponential fits to the data.

To further confirm the existence of these in‐plane charge transfer states, the PL of the nanoribbons was measured at cryogenic temperatures (**Figure** **5**a). Lowering the sample temperature to 5 K leads to a drastic narrowing of the PL spectrum, and two distinct spectral features become apparent. These include a very weak emission peak at ≈630 nm, which resembles that of the PMI‐L5 molecules measured at the same temperature (Figure S3, Supporting Information) and hence is assigned to Frenkel excitons or their excimers. The second feature is a more prominent, red‐shifted emission peak centered at ≈700 nm that can be attributed to the charge transfer states. The charge transfer origin of this 700 nm peak is further confirmed by time‐resolved PL measurements (Figure 5b). Due to the spatial separation of charge carriers in the ^1^CT_intra_ and ^1^CT_inter_ states, they would recombine more slowly than the Frenkel excitons, whose electrons and holes reside on the same molecules. The PMI‐L5 molecules, which predominantly host Frenkel excitons, exhibit an average lifetime of ≈5 ns (see Section S2, Supporting Information for fitting results). In contrast, the decay curves of the supramolecular nanoribbons show an additional slow component that extends the average lifetime to ≈14 ns, further confirming the contributions of the in‐plane charge‐transfer states to the emission.

### 2.4 Triplet Formation via Inter‐Column Charge Recombination

The spin‐uncorrelated charge carriers spatially separated in neighboring molecular columns can populate the inter‐column singlet ^1^CT_inter_ and triplet ^3^CT_inter_ states in a 1:3 ratio (Figure 5e). While the ^1^CT_inter_ recombination toward the singlet ground state (^1^S_0_) is spin allowed, the ^3^CT_inter_ to ^1^S_0_ transition is spin forbidden. Since the lowest triplet state (^3^T_1_) energy of the molecules, which is ≈1000 nm,^[^ ^35^, ^36^ ^]^ is much smaller than that of the ^3^CT_inter_ states (≈700 nm, almost degenerate in energy with ^1^CT_inter_),^[^ ^33^ ^]^ and the transition between these two triplet states is spin allowed, downhill relaxation of the inter‐column ^3^CT_inter_ to the molecular triplet state ^3^T_1_ is energetically favorable. Direct observation of phosphorescence from ^3^T_1_ is largely prohibited because of its long lifetime, although a weak tail extending to ≈1300 nm is indeed observed in the emission spectra of the nanoribbons at 5 K (Figure 5a inset), which may be associated to weak emission from ^3^T_1_.

Transient absorption (TA) measurements further confirm the efficient formation of triplet excitons in the nanoribbons. Figure 5c,d,f,g show room‐temperature TA spectra and kinetic traces of PMI‐L5 molecules and nanoribbons in water under vacuum conditions. Upon 440 nm femtosecond pulsed laser excitation, the PMI‐L5 molecules display a negative ground‐state bleaching band (^1^S_0_ → ^1^S_1_/excimer) at below 490 nm (Figure 5d). Strong positive excited state absorption bands (^1^S_1_/excimer → ^1^S_n_) with maxima at 530 and 630 nm that are likely associated with Frenkel/excimer states^[^ ^37^, ^38^ ^]^ can also be observed and they decay rapidly within ≈140 ps after the initial excitation (Figure 5g). TA of the nanoribbons exhibits several distinct features compared to that of the molecules. Positive excited state absorption bands at 520, 550, and 630 nm are observed (Figure 5c). The latter two exhibit long kinetic components apparently extending beyond the 3 ns time window of the experiment, suggesting the contributions of charge transfer states to these spectral features (Figure 5f, green), consistent with the emission lifetime observed in Figure 5b. The most notable feature in the nanoribbon spectra compared to the molecules is the emergence of an additional absorption band centered at 570 nm (Figure 5c, orange arrow). This band, with an average rise time of ≈120 ps, increases in concert with the decay of the 550 and 630 nm features and persists across the entire ≈3 ns time window (Figure 5f, orange). Extending the time window of the transient absorption spectra to 50 µs shows that this absorption feature exhibits negligible decay during the time window (Figure S5, Supporting Information). The peak position of this absorption band is also consistent with previously reported triplet formation features in TA measurements of PMI molecules.^[^ ^39^, ^40^ ^]^ We, therefore, assign this long‐lived feature to the ^3^T_1_ → ^3^T_n_ absorption, and its rising time signifies that filling of the triplet state (^3^T_1_) by the ^3^CT_inter_ states occurs within ≈100 ps after the initial optical pumping (corresponding to the gray arrow between the ^3^CT_inter_ and ^3^T_1_ states in Figure 5e).

From the measured optical transition dipoles, we can further estimate that of all the photogenerated charge carriers, only <1% of them recombine and emit through the Frenkel exciton state, while the rest undergo charge separation and transport predominantly along the 1D molecular columns until: i) ≈20% of them recombine through the intra‐column charge transfer state (^1^CT_intra_) radiatively or nonradiatively, ii) ≈20% of them recombine through the inter‐column singlet charge transfer state (^1^CT_inter_) radiatively or nonradiatively, and iii) ≈60% of them recombine and populate the inter‐column triplet states (^3^CT_inter_) (see Section S3, Supporting Information for estimation details). As such, the packing order of monomers in the nanoribbons effectively creates interfaces among adjacent molecular columns. This, combined with the large intra‐column charge mobility, promotes high triplet formation yields (overall ≈60%), a drastic increase from the <1% formation yield in PMI molecules generated through the ^1^S_1_ → ^3^T_1_ ISC process.^[^ ^36^ ^]^

## 3 Conclusions

In summary, we study the influence of lattice symmetry on the spin states and carrier recombination pathways in supramolecular polymers. In a supramolecular polymer with a 2D oblique crystalline lattice symmetry, we find that the preferential dissociation of charge carriers along the direction with a smaller intermolecular distance leads to efficient recombination of spin‐uncorrelated charge carriers along the perpendicular direction. This, as a result, leads to a prominent emission transition dipole along the direction with a larger intermolecular distance, and a substantial triplet formation yield. These findings elucidate the critical role played by lattice symmetry in the photophysical properties of self‐assembled supramolecular systems and have broader implications for various scenarios that involve triplet formation, whether desired or not. For instance, this concept of using lattice symmetry for isolating the charge transport and recombination processes could be particularly appealing for photon up‐conversion^[^ ^3^ ^]^ and photocatalysis,^[^ ^41^, ^42^ ^]^ where efficient and directional triplet formation at interfaces is essential.

## 4 Experimental Section

### Preparation of Supramolecular Nanoribbons

The chromophore amphiphile molecules, PMI‐L5 and PMI‐L5‐PA, used in this study were synthesized following previously reported methods.^[^ ^13^, ^20^ ^]^ To prepare the supramolecular nanoribbons, the synthesized PMI‐L5 molecules were dissolved in 10 mm NaOH aqueous solutions to a concentration of 10 mm, resulting in deep red solutions. Mixtures with varying molar ratios between PMI‐L5 and PMI‐L5‐PA were prepared by volumetrically mixing these two solutions. The resulting solutions were mixed with NaCl aqueous solutions (300 mm) at a volume ratio of 5:1 molecule solution:NaCl aqueous solution with vortexing, leading to an 8.3 mm PMI‐L5 solution. This solution was then annealed at 95 °C in a dry heating bath (14‐955‐241, Fisher Scientific) for 1 h, after which the dry bath was turned off and the samples slowly cooled over the next hour before being removed.

Supramolecular nanoribbon samples for PL measurements were prepared by diluting the annealed solutions ten‐fold using 50 mm NaCl aqueous solutions and drop casting 200 µL of the solution onto pre‐cleaned substrates. The solution was rested for 30 s before being wicked away and allowed to dry. For a sparser coverage, 1 mL of magnesium acetate aqueous solution (10 mm) was added to the substrate and then wicked away.

### Sample Characterizations

AFM measurements were performed on a Veeco MultiMode 8 scanning probe microscope with a NanoScope V controller (Veeco Instruments) operating in tapping mode and in air. The samples were prepared by drop casting 10 µL of 1 mm nanoribbon solutions onto precleaned silicon substrates. After drying, the samples were washed by dropping 10 µL DI water onto the substrate surface. The droplets were then gently wicked away from the edge. UV/Vis absorption spectra were recorded using a Cary‐500 (Varian) spectrometer.

### Optical Imaging and Spectroscopic Measurements

Real‐space and BFP imaging were performed on a home‐built confocal laser microscope allowing switching between 4f Fourier imaging and real‐space imaging configurations (see Figure S7 , Supporting Information for its layout). The samples were excited by a 440 nm laser. An oil objective (60x) with a numerical aperture (NA) of 1.35 was used to focus the laser beam onto the samples. The same objective was used to collect emission, which was directed to a 300 mm spectrometer equipped with an EMCCD. For polarization‐dependent measurements, a linear polarizer and a half‐wave plate were used to select the polarization of the excitation beam or the collected emission. Time‐resolved PL measurements were performed by sending collected emissions to single‐photon avalanche diodes. For low‐temperature optical measurements, the samples were loaded into a continuous‐flow liquid He cryostat and mounted onto the microscope. A long‐working‐distance microscope objective (40x, NA = 0.7) was used for focusing laser beams and collecting emissions at cryogenic temperatures.

### Transient Absorption Measurements

Ultrafast TA spectra and kinetics were carried out using an amplified Ti:sapphire laser system (Spectra Physics, Solstice Ace) and an automated data acquisition system (Ultrafast Systems, HELIOS). The amplifier produced 800 nm, 60 fs pulses at 5 kHz. The output from the amplifier was split 90:10 with 90% used to pump an optical parametric amplifier (Light Conversion, TOPAS) which provided the excitation pulses. A broadband continuum probe beam was generated after double‐passing the remaining 10% output from the laser amplifier down a computer‐controlled optical delay line and then focusing it into a 3 mm thick piece of sapphire. The residual 800 nm light was removed from the probe beam with a low‐pass interference filter leaving a 440–750 nm white‐light continuum. The probe beam was then focused into a stirred 2 mm quartz cuvette containing the solution to be measured. The transmitted probe beam was detected using a fiber optically coupled spectrograph with a 1 D, 2048‐pixel CCD array detector. The differential extinction ΔA = −log_10_(T_ON_/T_OFF_) was calculated by using a synchronous chopper to block every other excitation pulse. The excitation pulse beam (0.24–0.28 µJ pulse^−1^) was depolarized and then overlapped with the probe beam spot in a 2 mm path‐length fused silica cuvette. The temporal chirp in the probe pulse was measured and corrected by making a measurement on neat solvent; the resonant signal was then fitted for each probe wavelength to determine the zero‐delay position between the pump and probe. For pump‐probe delay intervals longer than 3 ns (up to 50 µs for this work), the probe light was instead generated by a continuum light source (Ultrafast Systems, EOS) triggered by the data acquisition software. A photodiode pick‐off and fast‐timing electronics were used to determine the pump‐probe delay time with 200 ps resolution.

### Monte Carlo Simulations

In the simulations, the supramolecular nanoribbons were represented by 2D lattices. Each molecule was modeled as a three‐level system: the Frenkel state “2”, the charge carrier state “+1” and “−1” for holes and electrons, respectively, and the ground state “0”. The time step, δ*t*, was set to be 10 ps, to ensure that it was sufficiently shorter than the exciton and charge carrier recombination lifetimes. The effective masses of electrons and holes were considered to be equal to the electron rest mass, while that of Frenkel excitons was set to be an order of magnitude higher since it is strongly localized and the effective‐mass approximation is not valid.^[^ ^43^ ^]^ Details about the parameters used in the simulations are listed in Section S4 (Supporting Information).

## Conflict of Interest

The authors declare no conflict of interest.

## Supporting information

Supporting Information

## Data Availability

The data that support the findings of this study are available from the corresponding author upon reasonable request.
