# Supplementary material for: Lattice Symmetry‐Guided Charge Transport in 2D Supramolecular Polymers Promotes Triplet Formation
Source: Adv Sci (Weinh). 2024 Jun 12;11(30):2402932. doi: 10.1002/advs.202402932 (PMC11321616; doi:10.1002/advs.202402932)
Supplement: Supplementary file 1 — Supporting Information [file ADVS-11-2402932-s001.pdf]

ADVANCED SCIENCE
Open Access

# Supporting Information

for *Adv. Sci.*, DOI 10.1002/advs.202402932

Lattice Symmetry-Guided Charge Transport in 2D Supramolecular Polymers Promotes Triplet Formation

*Ruggero Emmanuele, Hiroaki Sai, Jia-Shiang Chen, Darien J. Morrow, Luka Đorđević, David J. Gosztola, Saw Wai Hla, Samuel I. Stupp and Xuedan Ma**

# Supporting Information

**Lattice Symmetry-Guided Charge Transport in Two-Dimensional Supramolecular Polymers Promotes Triplet Formation**

Ruggero Emmanuele, Hiroaki Sai, Jia-Shiang Chen, Darien J. Morrow, Luka Đorđević, David J. Gosztola, Saw Wai Hla, Samuel I. Stupp, Xuedan Ma*

## S1. Simulations of back-focal-plane images

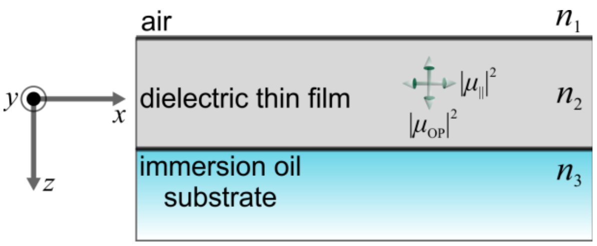


Figure S1. Side-view of the three-layer structure used in the simulation.

The formulism used for the simulations in this study were based on those developed by Schuller et al.[1] In principle, we consider a three-layer system including air (layer 1, refractive index $n_1$ = 1), supramolecule structures (layer 2, refractive index $n_2$ = 1.78), and immersion oil on substrate (layer 3, refractive index $n_3$ = 1.52) (Fig. S1). We assign the *a*- and *b*-axes of the supramolecule nanoribbons to be the *x*- and *y*-directions of the sample plane, and the direction perpendicular to the nanoribbon surface the *z*- or out-of-plane (OP) direction. For a nanoribbon with an overall emission transition dipole moment $\boldsymbol{\mu}$, whose dipole strength along the *x* (*a*), *y* (*b*), and *z* (OP) directions are $|\boldsymbol{\mu}_a|^2$, $|\boldsymbol{\mu}_b|^2$, and $|\boldsymbol{\mu}_{\mathrm{OP}}|^2$, its spontaneous decay rate (Γ) at electromagnetic frequency *ω* can be derived using the Fermi's golden rule:[1-3]

$$\Gamma^{s,p}_{l=a,b,\mathrm{OP}}(\omega, k_{\|}) = C_0(\omega)\left|\boldsymbol{\mu}_{l=a,b,\mathrm{OP}}(\omega)\right|^2 \tilde{\rho}^{s,p}_{l=a,b,\mathrm{OP}}(\omega, k_{\|}) \qquad (1)$$

with $C_0(\omega) = \frac{\omega^3}{c^3 \hbar \pi \varepsilon_0}$, and $\tilde{\rho}^{s,p}_{l=a,b,\mathrm{OP}}(\omega, k_{\|})$ being the vacuum-normalized local density of optical states. Here, $\varepsilon_0$ is the vacuum permittivity, $s$ and $p$ are the polarization of the electromagnetic field, $k_{\|}$ is the in-plane component of the momentum vector $\vec{k}$. The overall emission, *I*, projected at the ($k_x$, $k_y$) plane is then

$$I^{s,p}(\omega, k_{\|}) = \eta \sum_{l=a,b,\mathrm{OP}} \Gamma^{s,p}_{l}(\omega, k_{\|}) \qquad (2)$$

with $\eta$ being a constant related to the measurement condition.

The analytical expressions for the normalized local density of optical states, $\tilde{\rho}^{s,p}_{l=a,b,\mathrm{OP}}(\omega, k_{\parallel})$, can be derived using the reciprocity theorem:

$$\tilde{\rho}^{p}_{a}(\omega, k_{\parallel}) = \tilde{\rho}^{s}_{b}(\omega, k_{\parallel}) = \tilde{\rho}^{s}_{\mathrm{OP}}(\omega, k_{\parallel}) = 0 \qquad (3)$$

$$\tilde{\rho}^{s}_{a}(\omega, k_{\parallel}) = \left(\frac{1}{8\pi k_0^2}\right)\left(\frac{k_0}{k_{z3}}\right)\left|\frac{t^{s}_{32}e^{2ik_{z2}d}(1+r^{s}_{21}e^{2ik_{z2}d})}{1-r^{s}_{21}r^{s}_{23}e^{2ik_{z2}d}}\right|^2 \qquad (4)$$

$$\tilde{\rho}^{p}_{b}(\omega, k_{\parallel}) = \left(\frac{1}{8\pi k_0^2}\right)\left(\frac{k_0}{k_{z3}}\right)\left|\frac{t^{p}_{32}e^{2ik_{z2}d}\frac{k_{z2}}{n_2 k_0}(1-r^{p}_{21}e^{2ik_{z2}d})}{1-r^{p}_{21}r^{p}_{23}e^{2ik_{z2}d}}\right|^2 \qquad (5)$$

$$\tilde{\rho}^{p}_{\mathrm{OP}}(\omega, k_{\parallel}) = \left(\frac{1}{8\pi k_0^2}\right)\left(\frac{k_0}{k_{z3}}\right)\left|\frac{t^{p}_{32}e^{2ik_{z2}d}\frac{k_{\parallel}}{n_2 k_0}(1+r^{p}_{21}e^{2ik_{z2}d})}{1-r^{p}_{21}r^{p}_{23}e^{2ik_{z2}d}}\right|^2 \qquad (6)$$

Here, $d$ is the thickness of the supramolecular nanoribbon, $n_i$ is the refractive index in layer $i$, $t^{s,p}_{ij}$ and $r^{s,p}_{ij}$ represent $s$ and $p$ polarized transition ($t$) or reflection ($r$) coefficients from layer $i$ to layer $j$:

$$t^{p}_{ij} = \frac{2n_i n_j k_{zi}}{n_j^2 k_{zi}+n_i^2 k_{zj}},\ t^{s}_{ij} = \frac{2k_{zi}}{k_{zi}+k_{zj}},\ r^{p}_{ij} = \frac{n_j^2 k_{zi}-n_i^2 k_{zj}}{n_j^2 k_{zi}+n_i^2 k_{zj}},\ r^{s}_{ij} = \frac{k_{zi}-k_{zj}}{k_{zi}+k_{zj}} \qquad (7)$$

Using this model, we can construct BFP images and compare them with the experimentally measured ones to obtain the ratios among the transition dipole strength along the $a$, $b$, OP directions, $|\boldsymbol{\mu}_a|^2 : |\boldsymbol{\mu}_b|^2 : |\boldsymbol{\mu}_{\mathrm{OP}}|^2$.

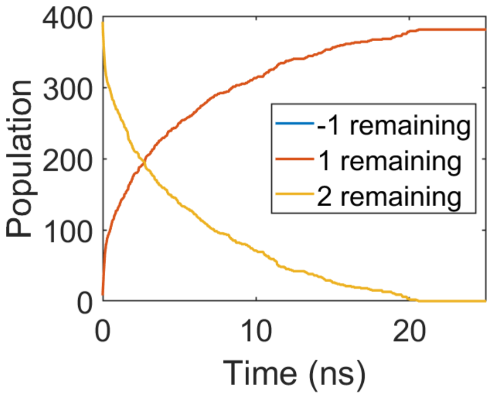


Figure S2. Population evolutions of Frenkel excitons (“2”) and charge carriers (“1” and “-1”) in a 220 x 60 2D lattice at various time delays after the initial optical excitation.

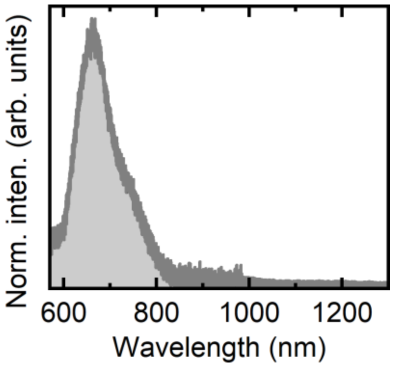


Figure S3. Photoluminescence spectrum of PMI-L5 molecules at 5 K.

## S2. Fitting of time-resolved photoluminescence decay curves

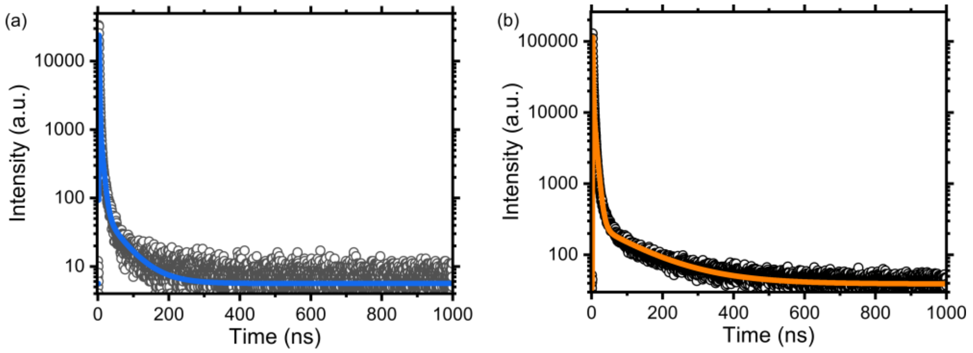


Figure S4. Decay curves (circles) and fitting results (curves) of molecules (a) and nanoribbons (b) measured at 5 K. The instrument response function is deconvoluted during the fitting. Fitting results are listed in the table below.

| | Amplitude 1 | $t_1$ (ns) | Amplitude 2 | $t_2$ (ns) | Amplitude 3 | $t_3$ (ns) | $t_{avg}$ (ns) |
|---|---|---|---|---|---|---|---|
| Molecules | 128789 | 1 | 4373 | 7 | 157 | 56 | 5 |
| Nanoribbons | 380119 | 1 | 29000 | 7 | 397 | 134 | 14 |

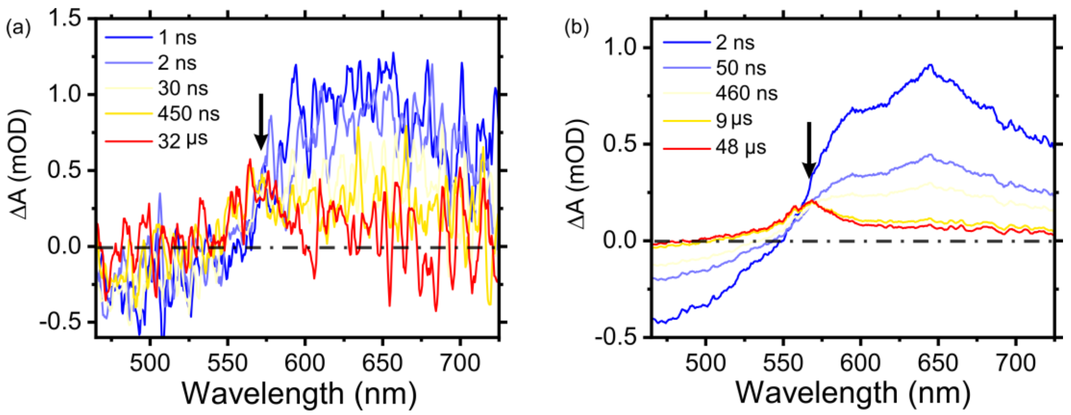


Figure S5. (a) Transient absorption spectra at selected pump-probe delays of nanoribbons upon 440 nm femtosecond laser excitation. (b) Decay spectra obtained by an SVD analysis.

## S3. Estimation of recombination pathways

If we assume that in a given time, the number of photons emitted by the Frenkel exciton state is $n_0$, based on the transition dipole ratios determined from the BFP studies ($|\boldsymbol{\mu}_a|^2 : |\boldsymbol{\mu}_b|^2 : |\boldsymbol{\mu}_{OP}|^2 = 6.0 : 11.6 : 1.0$), we can derive that photons emitted through the $^1CT_{intra}$ state and $^1CT_{inter}$ state are $6n_0$ and $12n_0$, respectively. Because quantum yield of the PMI molecule is close to unity,[4] we can assume that the $^1S_1 \rightarrow {}^1S_0$ recombination occurs predominantly radiatively with negligible nonradiative processes. However, quantum yields of the $^1CT_{intra}$ and $^1CT_{inter}$ states are prior unknown. We define them as $^1QY_{intra} = 6n_0/(6n_0 + n_b)$ and $^1QY_{inter} = 12n_0/(12n_0 + n_a)$, respectively, with $n_b$ and $n_a$ being the corresponding numbers of carrier pairs recombined nonradiatively. For simplicity, we assume $n_b \approx n_a$ because of the long lifetimes of the $^1CT_{intra}$ and $^1CT_{inter}$ states. Since the $^1CT_{inter}$ and $^3CT_{inter}$ states are populated in a 1:3 ratio, we can determine that populations of carrier pairs recombined through the $^3CT_{inter}$ state is $3(12n_0 + n_a)$, most of which would relax to the molecular triplets or recombine nonradiatively. Taken together, the overall quantum yield of the nanoribbons can be written as $QY = 19n_0/(19n_0 + n_b + n_a + 3(12n_0 + n_a))$.

The supramolecular nanoribbons have an overall quantum yield of ~ 1%.[5] We can thus derive that percentages of charge carriers that recombine through the $^1S_1$, $^1CT_{intra}$, $^1CT_{inter}$ and $^1CT_{inter}$ states to be 0.05%, 20%, 20%, and 60%, respectively.

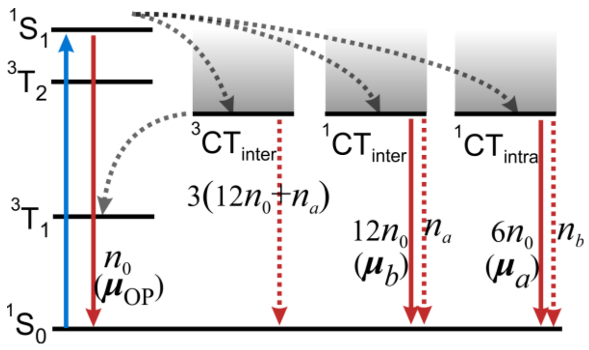


Figure S6. Energy levels and carrier populations involved in the estimation. Solid and dashed red arrows represent radiative and nonradiative recombination, respectively.

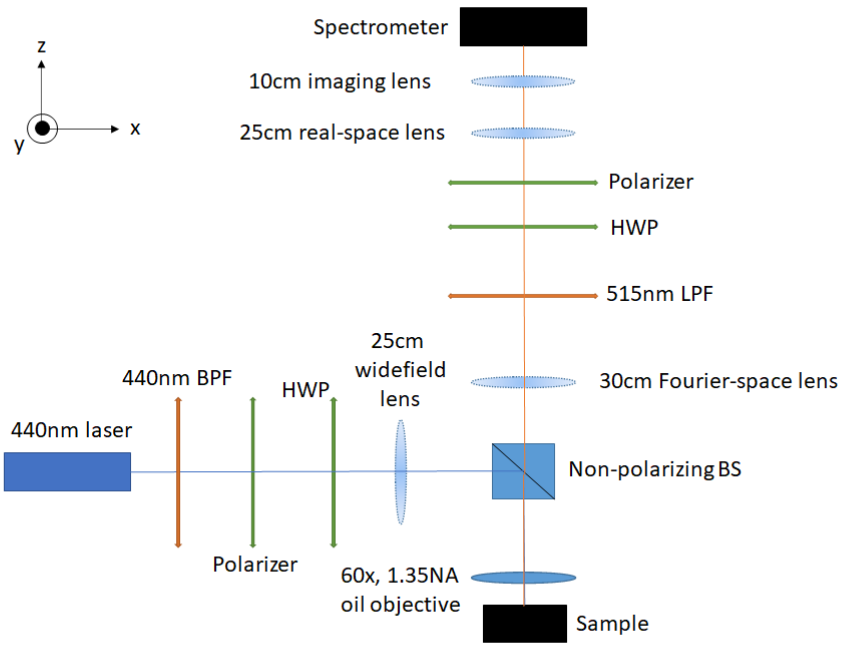


Figure S7. Sketch of the optical microscope used for real-space and BFP imaging and spectroscopy. BPF: band-pass filter; HWP: half-wave plate; BS: beam splitter; LPF: long-pass filter. The lenses with dashed lines are removable, allowing a switching between the real-space and BFP imaging mode.

## S4. Parameters used in the Monte Carlo simulations

Table S1. Details of parameters used in the Monte Carlo simulations

| Parameters | Values (units) | Notes |
|---|---|---|
| $\Gamma_{ET}$ | 2 $ns^{-1}$ | Derived from Ref. 6,7 |

| | | |
|---|---|---|
| $\Gamma_{CT}$ | 4 $ns^{-1}$ | Derived from Ref. 6,7 |
| $d$ | 3.6 (Å) along *a*-axis<br>8.3 (Å) along *b*-axis | Ref. 8 |
| $\Delta E$ | 600 ($cm^{-1}$) for Frenkel excitons<br>625 ($cm^{-1}$) for charge carriers (electrons and holes) | Ref. 9 |
| $m$ | 10 $m_0$ for Frenkel excitons<br>$m_0$ for charge carriers | $m_0$ = electron mass at rest |
| $\delta t$ | 0.01 ns | |
| $k_{FE}$ | 1 $ns^{-1}$ | Decay rate of the Frenkel excitons; estimated from room temperature time-resolved photoluminescence measurements |
| $k_{CTE}$ | 0.2 $ns^{-1}$ | Decay rate of the charge carriers; estimated from room temperature time-resolved photoluminescence measurements |
| $k_{dissociation}$ | 20 $ns^{-1}$ | Derived from Ref. 9 |

Due to the lack of previously reported $\Delta E$ values for this system, the values adapted here are mostly relative values between the Frenkel excitons and charge carriers based on their respective matrix elements that facilitate their hopping to neighbors.[9] Rectangular barriers are considered and the WKB approximation is used.

Reference


1 J. A. Schuller, S. Karaveli, T. Schiros, K. He, S. Yang, I. Kymissis, J. Shan, R. Zia. Orientation of luminescent excitons in layered nanomaterials. *Nat. Nanotechnol.* **8**, 271-276 (2013).

2 T. H. Taminiau, S. Karaveli, N. F. van Hulst, R. Zia. Quantifying the magnetic nature of light emission. *Nat. Commun.* **3**, 979 (2012).

3 L. Peng, X. Wang, I. Coropceanu, A. B. Martinson, H. Wang, D. V. Talapin, X. Ma. Titanium Nitride Modified Photoluminescence from Single Semiconductor Nanoplatelets. *Adv. Funct. Mater.* **30**, 1904179 (2020).

4 T. Kircher, H.-G. Lohmannsroben. Photoinduced charge recombination reactions of a perylene dye in acetonitrile. *Phys. Chem. Chem. Phys.* **1**, 3987-3992 (1999).

5 A. S. Weingarten, R. V. Kazantsev, L. C. Palmer, M. McClendon, A. R. Koltonow, A. P. S. Samuel, D. J. Kiebala, M. R. Wasielewski, S. I. Stupp. Self-assembling hydrogel scaffolds for photocatalytic hydrogen production. *Nat. Chem.* **6**, 964-970 (2014).

6 L. Peng, X. Ma, H. Zhu, O. Chen, W. Wang. Influence of local structures on the energy transfer efficiencies of quantum-dot films. *Phys. Rev. B* **102**, 035437 (2020).

7 Y. Gao, C. S. S. Sandeep, J. M. Schins, A. J. Houtepen, L. D. A. Siebbeles Disorder strongly enhances Auger recombination in conductive quantum-dot solids. *Nat. Commun.* **4**, 2329 (2013).

8 A. S. Weingarten, R. V. Kazantsev, L. C. Palmer, D. J. Fairfield, A. R. Koltonow, S. I. Stupp. Supramolecular Packing Controls H2 Photocatalysis in Chromophore Amphiphile Hydrogels. *J. Am. Chem. Soc.* **137**, 15241-15246 (2015).

9 N. J. Hestand, R. V. Kazantsev, A. S. Weingarten, L. C. Palmer, S. I. Stupp, F. C. Spano. Extended-Charge-Transfer Excitons in Crystalline Supramolecular Photocatalytic Scaffolds. *J. Am. Chem. Soc.* **138**, 11762-11774 (2016).
